# Emergency Room Validation of the Revised Suicide Trigger Scale (STS-3): A Measure of a Hypothesized Suicide Trigger State

**DOI:** 10.1371/journal.pone.0045157

**Published:** 2012-09-14

**Authors:** Zimri S. Yaseen, Evan Gilmer, Janki Modi, Lisa J. Cohen, Igor I. Galynker

**Affiliations:** 1 Department of Psychiatry and Behavioral Sciences, Beth Israel Medical Center, New York, New York, United States of America; 2 Department of Psychiatry and Behavioral Sciences, Albert Einstein School of Medicine, Beth Israel Medical Center, New York, New York, United States of America; The University of Hong Kong, Hong Kong

## Abstract

**Background:**

The Suicide Trigger Scale (STS) was designed to measure the construct of an affective ‘suicide trigger state.’ This study aims to extend the inpatient setting validation study of the original Suicide Trigger Scale version 2 to the revised Suicide Trigger Scale version 3 (STS-3) in an acute psychiatric emergency room setting.

**Methods:**

The 42-item STS-3 and a brief psychological test battery were administered to 183 adult psychiatric patients with suicidal ideation or attempt in the psychiatric emergency room, and re-administered to subjects at 1 year follow up. Factor analysis, linear and logistic regressions were used to examine construct structure, divergent and convergent validity, and construct validity, respectively.

**Results:**

The STS-3 demonstrated strong internal consistency (Cronbach’s alpha 0.94). Factor analysis yielded a three-factor solution, which explained 43.4% of the variance. Principal axis factor analysis was used to identify three reliable subscales: Frantic Hopelessness, Ruminative Flooding, and Near-Psychotic Somatization (Cronbach’s alphas 0.90, 0.80, and 0.76, respectively). Significant positive associations were observed between *Frantic Hopelessness* and BSI depression and anxiety subscales, between *Ruminative Flooding* and BSI anxiety and paranoia subscales, and Near Psychotic Somatization and BSI somatization subscales. Suicidal subjects with suicide attempt history had mean scores 7 points higher than those without history of suicide attempts. Frantic hopelessness was a significant predictor of current suicide attempt when only attempts requiring at least some medical attention were considered.

**Conclusion:**

The STS-3 measures a distinct clinical entity, provisionally termed the ‘suicide trigger state.’ Scores on the STS-3 or select subscales appear to relate to degree of suicidality in terms of severity of ideation, history of attempt, and presence of substantive current attempts. Further study is required to confirm the factor structure and better understand the nature of these relations.

## Introduction

Suicide is estimated to be the 10^th^ leading cause of death in the United States, and ranges between second and fourth leading cause for age groups between 10 and 54 years of age [1], making suicide an important public health problem. While a wide body of literature exists on the chronic risk factors of suicide [2], [3] the acute factors that lead a person to make a suicide attempt (SA) remain inadequately understood. Because of the difficulty in predicting imminent suicidal risk, still no instruments exist which can reliably identify persons who will make a suicide attempt in the near future [4].

A study by Deisenhammer et al. [5] reported that the transition from the first emergence of suicidal ideation to actual suicide attempts may be as short as ten minutes, leaving clinicians a very small window for detection and intervention. It was hypothesized that in susceptible individuals this transition from suicidal ideation to attempt may be marked by a distinct psychopathological pre-suicidal state [6]–[8]. Results from studies conducted by Fawcett, et al. [6] indicate that suicidal individuals often experience increased anxiety and agitation, a combination of symptoms which he has termed “psychic pain”, before a serious suicide attempt. Further, in a series of studies, Hendin and colleagues [7], [9]–[11] have reported that an acute, high-intensity, negative affect state - a time-limited suicide crisis - served as a trigger for SA. In agreement with this hypothesis, a number of other reports have linked increased risk for suicide with affectively intense panic attacks [12]–[14], while a recent study by Katz, et al., [15] hypothesized that panic attacks may serve as a mediator for transition from suicidal ideation to suicide attempt in depressed patients.

At present, there exist no instruments designed to capture the phenomenology of the suicide crisis – a suicide trigger state, indicating the risk of imminent suicide [2], [6]. In their absence, clinicians must rely on well-known factors indicating chronic suicide risk such as suicidal ideation, history of suicide attempts, severe psychopathology, history of psychiatric hospitalization, substance abuse, and poor social supports [2], [3]. Of note, while some scales for the assessment of chronic suicide risk have been validated, including the Suicide Assessment Scale [16], Suicide Intent Scale [17] and Motto and Bostrom’s proposed scale [18] others, such as the SAD PERSONS scale [19] exhibited clinically limited predictive validity when tested [20]. Research and clinical work in suicidology is further complicated by lax terminology combining ideation, serious attempts with the intent to die, gestures, and deliberate self harm under a broad term of “suicidality.” In this regard, recent adoption of the Columbia Suicide Severity Rating Scale (C-SSRS) as a standard for identifying and quantifying both suicidal ideation and suicide attempts represents a major advance in suicide research and clinical work [21].

In our previous work on defining and measuring a distinct, acute, pre-suicidal state we have identified a panic-like psychopathological state found in suicide attempters, consistent with previous findings from Fawcett and Hendin [8], [11], [22]. This distinct and novel clinical entity, speculatively termed the “suicide trigger state” was identified and measured with the Suicide Trigger Scale, version 2 (STS-2), administered to 141 psychiatric inpatients. To reduce response bias by those wanting to either hide or exaggerate their suicidality, the STS-2 did not contain questions about suicidal ideation or intent. Nonetheless, STS-2 scores were significantly associated with reported lifetime history of suicide attempt.

Principal component analysis of STS-2 yielded a two-component solution. The first component described a combination of near psychotic somatization and ruminative flooding, while the second described frantic hopelessness. Based on item response theory analysis of the STS-2, reverse scored items on the STS-2 were converted to equivalent non-reverse score items in its revised form, the STS-3, and one item with poor performance (feeling that head or body parts had changed in size or shape) was removed. In addition, based on clinical analysis and observation, four new items assessing feelings of hopeless, entrapment, and psychic pain were added.

Thus in the present study we seek to replicate concurrent validity of the scale in the psychiatric emergency room setting, and refine our understanding of the scale structure by examining the revised scale on a larger sample size. We hypothesize that the STS-3 will demonstrate high internal consistency equivalent to the STS-2, and that ‘ruminative flooding’ and ‘near psychotic somatization’ constructs which did not separate in principal components analysis of the STS-2 would resolve as separate factors in exploratory factor analysis of the STS-3. Further, we hypothesize that higher scores on the STS-3 will be associated with higher rates of past, current, and future suicide attempts. Better understanding of the psychometric properties of the scale in different treatment settings may help further understanding of its best clinical application.

## Methods

### Participants

The study was approved by the Beth Israel Medical Center (BIMC) Institutional Review Board. All participants were interviewed in the BIMC Psychiatric Emergency Room. Males and females between 18 to 65 years of age, presenting with suicidal ideation or attempt, and able to understand and willing to sign the informed consent were included in the study. Patients exhibiting mental retardation, cognitive impairment, or linguistic limitations precluding understanding of the consent or research questions, or significant medical or neurological disease or possible delirium were excluded from the study. A total of 183 patients qualified for study inclusion, agreed to participate, signed all necessary consent and research authorization forms, and provided sufficient information to the researchers for use in the study.

### Instruments

#### Columbia Suicide Severity Rating Scale (C-SSRS)

The C-SSRS was developed by Posner et al. [23] and is a semi-structured interview for the identification and assessment of suicidal ideation and behavior. This scale has proven reliable and valid with inter-rater reliability rates close to 90%. The C-SSRS was approved by the FDA for use in all pharmaceutical drug trials. In addition to assessment of suicidal behavior ranging from preparatory acts to suicide attempt (distinguished from aborted and interrupted attempts), the C-SSRS produces a suicidal ideation severity rating ranging from a score of 0 (no ideation present) to 5 (active ideation with plan and intent). In our study, suicide ideation was rated according to the 0–5 Suicide ideation severity scale. *Current Attempt*. Suicide attempt was defined as a ‘yes’ rating on the C-SSRS item ‘potentially injurious act committed with at least some wish to die as a result of this act.’ Subjects were assessed for the presence of an *Actual Suicide Attempt* at the time of presentation to the psychiatric emergency room by reconciliation of subject report in the C-SSRS and their history of present illness as recorded in their emergency room charts. *Actual Attempt Lethality*. Actual suicide attempt lethality is rated in the C-SSRS on a 0–5 scale, from no or very minor injury requiring no care (0), to mild injury such as might be treated by first aid measures (1) to moderate, requiring some medical care (2), to moderate-severe injury, requiring hospitalization (3), to severe, requiring intensive care (4) to death (5). [21]. The actual lethality level of current attempts was assessed using the C-SSRS supplemented by information from patient charts. *Substantive Current Attempt*. For purposes of data analysis we defined subjects presenting with a *Current Attempt* with *Actual Attempt Lethality* ≥2 as having made a substantive current attempt.

#### Suicide Trigger Scale Version 3 (STS-3)

The STS-3 is a 42-item scale containing three response categories (0  =  not at all, 1  =  somewhat, 2  =  a lot). To avoid over-reliance on self-reported suicidal symptoms and reduce possibility of over- and underreporting of such symptoms, the scale does not contain questions overtly related to suicide. This version of the scale was derived from the STS-2, a 39-item questionnaire which previously demonstrated excellent internal consistency (Cronbach’s alpha of 0.95.) and a significant association with history of suicide attempt in a demographically-similar, inpatient psychiatric sample [8].

#### The Brief Symptom Inventory (BSI) [24]

The BSI is a brief, 53-item, psychological self-report symptom scale that is easy to administer in a psychiatric emergency room setting. It measures a broad range of psychopathology, yields nine subscales, and exhibits good internal consistency (subscale Cronbach’s alphas ranging 0.71–0.85).

### Procedure

#### Primary contact

Charts of patients currently in the BI psychiatric emergency room were reviewed for clinical records of suicidal ideation or attempt (SI or SA). Following identification of suicidal patients through charts, clinical staff was approached to confirm patients’ appropriateness for the study with regard to inclusion and exclusion criteria. Those found appropriate were approached by trained research assistants, explained the purpose of the study, informed that their participation in the study would aid in the development of a questionnaire used to measure emotional states, and asked if they were willing to participate. Following the study explanation, assistants detailed the risks and benefits of study participation along with participants’ rights. Participants then read and signed informed consent and research authorization forms. At the end of each interview, which lasted 30 minutes to an hour, patients were reimbursed $25 for their participation.

The patient provided demographic information during the initial interview while clinical diagnostic information was obtained from the patient’s clinicians and their charts. Patients were included in the substance use subgroup if the psychiatrist in the ER assessed them to have an active substance use disorder or current/recent psychoactive substance use with a suspected active substance use disorder other than tobacco/nicotine abuse or dependence.

The STS-3 was administered first because, as the scale does not contain questions overtly related to the subject of suicide, this order reduces the likelihood of biased answers commonly associated with other self-report suicide measures [25], [26]. The C-SSRS and BSI questionnaires followed administration of the STS-3.

#### One-year follow-up

Of 183 subjects, 36 were reachable after 12 months. Of the147 that were not reachable, 18 had their telephones disconnected, 25 had wrong numbers, 41 did not answer at least 5 telephone calls and did not respond to telephone messages, 32 subjects did not provide any contact information, 14 were homeless, and 6 were either in a rehabilitation program at the time or had moved to another state. Of the 36 that were reachable, 6 refused to participate, 10 agreed to be re-interviewed but did not come for a follow-up appointment, and 20 agreed to and participated in the follow-up interview. Of these, two subjects provided demographic and clinical information that was incompatible with the initial interview and were excluded from the study.

### Data Analysis

#### Reliability and internal structure

As a modified version of the STS was investigated on a new sample population, an exploratory factor analysis approach was chosen. [27] Internal structure was assessed using Principal axis factor analysis with Varimax optimized factor rotation with Kaiser normalization. A factor-loading threshold of >0.475 was selected to optimally assign items uniquely to subscales. Reliability for the scale and derived subscales was assessed by Cronbach’s alpha. Individual items were assessed by examination of their impact on Cronbach’s alpha.

#### Convergent and divergent validity

To control for significant intercorrelations between BDI subscales, the subscale scores of the Brief Symptom Inventory (BSI) were regressed against each STS-3 subscale in three multivariate regression models to identify the independent associations of STS-3 symptom domains with BSI related domains, and their lack of independent association with unrelated BSI domains. Each regression model comprised the nine BSI subscales as predictor variables regressed against each STS subscale as the outcome variable. In addition, partial correlations between each STS-3 subscale and each BSI subscale were calculated controlling for the remaining BSI subscales. Finally, Cronbach’s alphas were calculated for each BSI subscale as an additional check on the reliability of the BSI subscales for the study population.

#### Concurrent validity

Correlations between STS-3 total and subscale scores and C-SSRS severity of suicidal ideation score were calculated. Additionally, total and subscale score means were compared by independent groups t-test comparing subjects with and without C-SSRS defined history of actual attempt (including past and current attempts). Further, in order to account for variability in the severity of actual suicidality of subjects presenting to the psychiatric emergency room, separate binary logistic regressions of total and subscale scores were performed with three levels of suicidality as outcome variables: 1) C-SSRS defined history of suicide attempt, 2) C-SSRS defined current suicide attempt, and 3) C-SSRS defined current substantive suicide attempt. In a secondary exploratory analysis based on the results of the preceding analysis, an alternative scoring of the STS-3 was tested using receiver operator characteristic analysis as a discriminator between subjects presenting with substantive attempts versus subjects who did not.

#### Predictive validity

Predictive validity was assessed using Fisher’s Exact Test comparing direction of change in STS-3 total score among subjects reporting a C-SSRS-defined suicide attempt during the 1 year follow-up period and those who did not make an attempt during the same period.

All statistical tests were performed using the Statistical Package for the Social Sciences, version 16 (SPSS 16.0).

## Results

### Sample Characteristics

The 42-item STS questionnaire was administered to 183 patients. Table 1 details the demographic and clinical characteristics of the study population. Of all subjects, 62.8% were male, and 53% had a history of alcohol or substance abuse. All subjects had some degree of acute suicidal ideation. Of all subjects, 77% had history of suicide attempt (past or current), 20.2% were current attempters, and of those approximately half had made a substantive suicide attempt.

**Table 1:** Sample Characteristics.

Gender	Frequency	Percent
Male	115	62.8
Female	67	36.6
Other (M to F transsexual)	1	0.5
**Race**	**Frequency**	**Percent**
American Indian	4	2.2
Asian	4	2.2
African American	52	28.4
Caucasian	69	37.7
Other	39	21.3
**Severity of Suicidal Ideation**	**Frequency**	**Percent**
Wish to be Dead	14	7.7
Nonspecific Active Suicidal Thoughts	21	11.5
Active SI w/Method	27	14.8
SI w/Some Intent	37	20.2
SI w/specific plan & intent	82	44.8
**History of Suicide Attempts**	**Frequency**	**Percent**
None	42	22.9
Past only	104	56.8
Current	37	20.2
Substantive current	18	10.3
**Primary Axis 1 Dx**	**Frequency**	**Percent**
Psychotic	35	19.1
Bipolar No Psychosis	28	15.3
Unipolar + Anxiety	93	50.8
No Axis 1 Dx	28	15.3
**Substance Abuse**	**Frequency**	**Percent**
None	86	47
Alcohol and/or Drugs	97	53

### Reliability and Internal Structure

The STS-3 scale exhibited high internal consistency with a Cronbach’s alpha of 0.942 (n = 173). Average inter-item correlation was 0.280, and scale-length invariant rho was fair to good at 0.279 [28]. No item increased Cronbach’s Alpha when excluded. Item-total score correlations ranged from 0.377 to 0.668.

**Figure 1:**
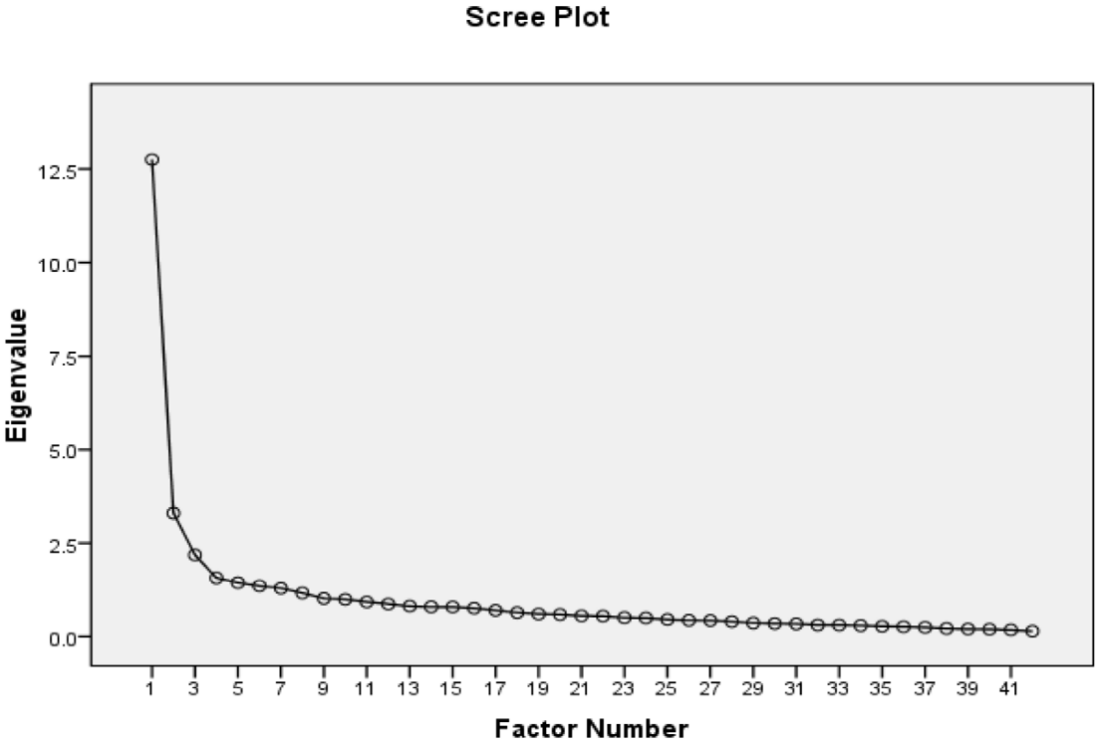
Scree plot for principal axis factor analysis. Figure 1 shows the scree plot for the principal axis factor analysis of the STS-3. Eigenvalues of the extracted factors are shown on the y-axis, while the ordinal factor number is shown on the x-axis.

Factor analysis (FA) using principal axis factor extraction on the 42-item STS-3 questionnaire extracted nine components with eigenvalues>1. The scree plot suggested a 3-factor solution (see Figure 1) accounting for 43.4% of the variance. Bartlett’s test of sphericity was significant (p<0.0005) and the Keyser-Meyer-Olkin measure of sampling adequacy was strong at 0.900. FA using principal axis extraction was repeated with restriction to a three-factor solution using Varimax rotation with Kaiser normalization. Using a factor-loading>0.475 as a cut-score to maximize factor coherence and exclude items loading similarly on multiple factors, three STS-3 subscales were derived from the rotated factors: Frantic Hopelessness, Ruminative Flooding, and Near Psychotic Somatization (see Table 2). Frantic hopelessness comprised 12 items describing entrapment, dread, and hopelessness, while ruminative flooding comprised 10 items describing incessant and overwhelming rumination, and near psychotic somatization comprised 7 items describing strange somatic experience and altered sensorium. These subscales demonstrated high internal reliability with Cronbach’s alphas of 0.906, 0.865, and 0.797, respectively. All items reduced Cronbach’s alpha when they were excluded from their subscale. Principal components analysis of each subscale with examination of scree plot demonstrated a single component for each subscale with all items loading strongly on that component (loading coefficients across all items and subscales ranged from 0.572 to 0.823).

**Table 2:** Individual STS item factor loadings for 3 factor solution and derived subscales.

STS Item:	Item #	Factor 1	Factor 2	Factor 3	Subscale: alpha	Subscale Scores: Mean[SD]
**no escape**	sts38	**0.768**	0.206	0.116	**Frantic Hopelessness**: 0.906	18.29[5.56]
**doom**	sts31	**0.720**	0.237	0.196		
**helpless to change**	sts26	**0.710**	−0.025	0.053		
**no way out**	sts12	**0.683**	0.228	0.112		
**will never change**	sts36	**0.670**	0.164	0.189		
**never be normal again**	sts24	**0.646**	0.074	0.156		
**dread**	sts29	**0.633**	0.115	0.183		
**trapped**	sts27	**0.613**	0.185	0.212		
**no solutions**	sts42	**0.610**	0.200	0.191		
**lost control to improve**	sts40	**0.538**	0.164	0.221		
**hopeless**	sts11	**0.527**	0.297	−0.007		
**no exit**	sts4	**0.513**	0.309	0.053		
expect the worst	sts18	0.464	0.361	0.05		
world closing in	sts15	0.446	0.367	0.274		
something horrible will happen	sts37	0.438	0.380	0.180		
hard to stop worrying	sts10	0.429	0.419	−0.013		
can’t think because too many thoughts	sts13	0.411	0.185	0.207		
unbearable inner pain	sts6	0.369	0.329	0.104		
inner pain must stop	sts33	0.352	0.301	0.151		
**headache from too many thoughts**	sts41	0.003	**0.676**	0.215	**Ruminative Flooding**: 0.865	15.01[4.56]
**head pressure from thinking too much**	sts39	0.145	**0.653**	0.319		
**can’t stop upsetting thoughts**	sts35	0.321	**0.589**	0.134		
**head explode from too many thoughts**	sts7	0.069	**0.575**	0.293		
**many thoughts**	sts3	0.212	**0.544**	0.076		
**can’t sleep because thoughts**	sts14	0.116	**0.536**	0.183		
**racing thoughts**	sts21	0.204	**0.533**	0.298		
**ideas turning over and over**	sts30	0.394	**0.514**	0.177		
**thoughts confused**	sts2	0.291	**0.483**	0.117		
**thoughts won’t go away**	sts34	0.318	**0.482**	0.120		
suddenly frightened	sts17	0.149	0.457	0.390		
wake up tired	sts1	0.266	0.401	0.140		
worry bad things may happen	sts9	0.206	0.318	0.129		
**something happing to body**	sts20	0.048	0.125	**0.788**	**Near Psychotic** **Somatization**: 0.797	5.59[3.73]
**strange sensations**	sts19	0.076	0.144	**0.643**		
**thing look strange**	sts8	−0.016	0.349	**0.546**		
**indescribable sensations**	sts25	0.189	0.127	**0.542**		
**felt blood rushing in veins**	sts28	0.058	0.296	**0.523**		
**unusual sensations**	sts5	0.21	0.119	**0.499**		
**something physically wrong**	sts32	0.207	0.059	**0.476**		
world is different	sts16	0.243	0.151	0.475		
senseless thoughts	sts23	0.163	0.236	0.353		
no control	sts22	0.308	0.341	0.348		

Extraction Method: Principal Axis Factoring. Rotation Method: Varimax with Kaiser Normalization.

As expected, given the high consistency of the STS-3 as a whole, subscale scores were significantly intercorrelated, but correlations were not so high as to make them redundant: Frantic Hopelessness correlated with Ruminative Flooding (r = 0.529, p<0.0005) and Near Psychotic Somatization (r = 0.378, p<0.0005), while Ruminative Flooding correlated with Near Psychotic Somatization as well (r = 0.514, p<0.0005). Each subscale was also strongly correlated with the STS total score (r = 0.811, 0.833, 0.709, p<0.0005, respectively).

### Convergent and Discriminant Validity

Stepwise forward linear regression revealed a significant positive association between *Frantic Hopelessness* and BSI subscales of depression (beta = 0.281, p = 0.007) and anxiety (beta = 0.274, p<0.009). Partial correlations were 0.231, p = 0.007, and 0.203, p = 0.018, respectively. No other subscales had significant partial correlations. *Ruminative Flooding* exhibited significant association with BSI subscales of anxiety (beta = 0.415, p<0.005) and paranoia (beta = 0.173, p = 0.036). Partial correlations were 0.291, p = 0.001, and 0.197, p = 0.022, respectively. No other subscales had significant partial correlations. *Near Psychotic Somatization* was found to be significantly associated with BSI subscales somatization, (beta = 0.485, p<0.0005) and phobia (beta = 0.221, p<0.023) and inversely correlated with depression (beta = −0.282, p = 0.005). Partial correlations were 0.316, p<0.0005, 0.134, p = 0.122, and −0.229, p = 0.008, respectively. No other subscales had significant partial correlations.

The STS-3 subscales did not associate with other unrelated symptom domains assessed by the BSI. BSI subscales demonstrated good internal reliability similar to published values, with Cronbach’s alpha ranging from 0.694 to 0.856: BSI Somatization 0.833, Obsession-Compulsion 0.794, Interpersonal Sensitivity 0.694, Depression 0.856, Anxiety 0.829, Hostility 0.830, Phobia 0.8.08, Paranoia 0.728, Psychoticism 0.696.

Further, the STS-3 total and subscale scores did not differ across any demographic or diagnostic subgroups. This suggests the STS scale measures a state that is not significantly influenced by demographic characteristics, and is distinct from personality, mood, and psychotic disorders.

### Concurrent Validity

All subjects were acutely suicidal in that they reported active suicidal ideation. STS-3 Total, Frantic Hopelessness, and Ruminative Flooding scores were significantly correlated with C-SSRS severity of ideation scores (r = 0.327, r = 0.323, and r = 0.289, p<0.0005, respectively).

The STS-3 score distribution had a mean of 56, corresponding to the previously published threshold score of 48 on the STS-2 for discriminating subjects with a history of suicide attempt (Yaseen et al., 2010), plus a maximum score on the 3 added items in the STS-3. Mean total score for subjects with history of suicide attempt was 59.5 versus 52.6 for those without. The difference was significant with two-tailed p = 0.026 (equal variances not assumed, Levene’s test for equal variances p = 0.016). Mean score on the Ruminative Flooding subscale for subjects with history of suicide attempt was 15.7 versus 13.9 for those without. The difference was significant with two-tailed p = 0.043 (equal variances not assumed, Levene’s test for equal variances p = 0.16). For Frantic Hopelessness a marginally significant difference in mean score was found 18.8 versus 16.8, two tailed p = 0.067 (equal variances not assumed, Levene’s test for equal variances p = 0.08). No significant difference was found for Near Psychotic Somatization. These results were replicated in univariate logistic regression, and when gender and substance abuse were added as covariates these effects were essentially unchanged. These effects though significant were quite small however: AORs ranging from 1.03 to 1.08.

Though neither the STS-3 total nor any of its subscale scores significantly associated with current attempt status, when analysis was restricted to substantive attempts (attempts with C-SSRS actual lethality of 2 or greater, i.e., requiring at least some medical attention beyond first-aide), Frantic Hopelessness was a significant predictor (AOR 1.165, p = 0.031) of current attempt and Near Psychotic Somatization was a significant protective factor, (AOR 0.846, p = 0.040, respectively). When gender and presence of substance abuse were added to the model these effects were slightly strengthened. Further, when BSI depression, BSI anxiety, and BSI item 32 ‘thoughts of death’ were added as potential predictor variables to the model, results were again essentially unchanged: Frantic Hopelessness was a significant predictor (AOR 1.222, p = 0.014) of current attempt and Near Psychotic Somatization was a marginally significant protective factor, (AOR 0.851, p = 0.060). Female gender also remained a positive predictor of suicide attempt (AOR 3.176, p = 0.039).

**Figure 2:**
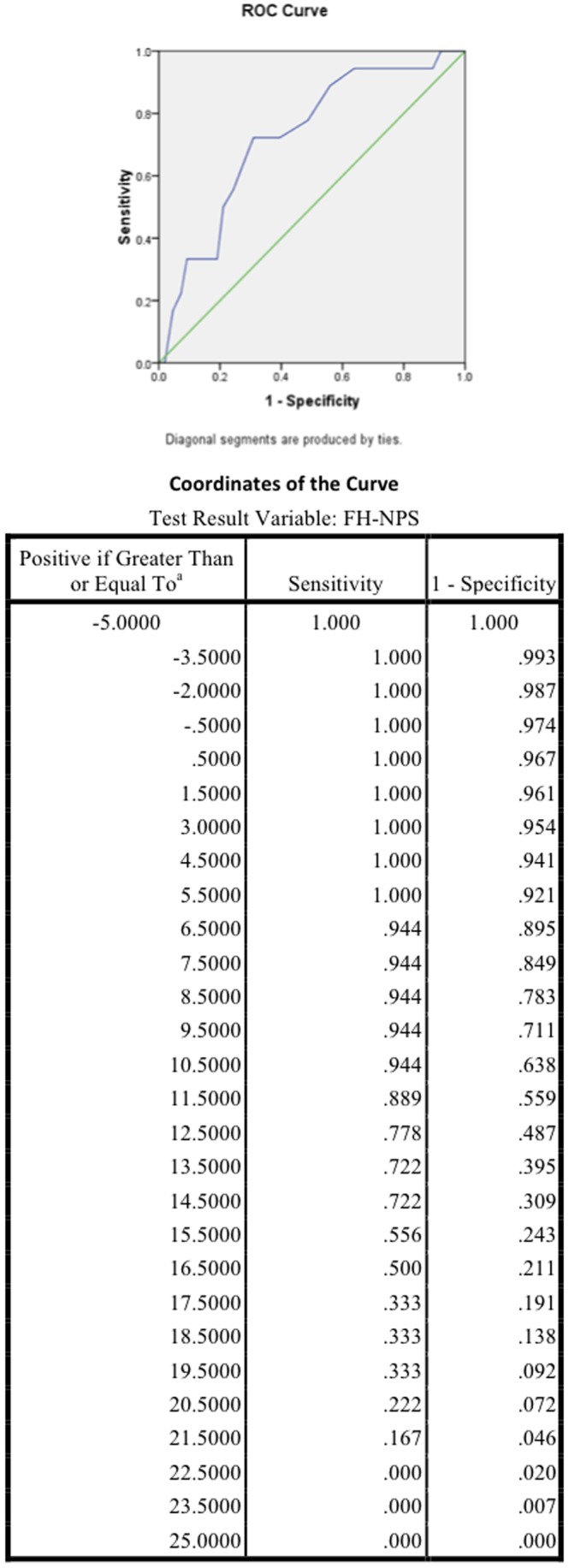
ROC curve for Frantic Hopelessness minus Near Psychotic Somatization (FH-NPS) as a predictor of substantive current suicide attempt. This post hoc analysis shows the Receiver Operator Characteristic curve and its coordinates for the FH-NPS score derived by subtracting scores on the “Near Psychotic Somatization” subscale of the STS-3 from scores on the “Frantic Hopelessness” subscale of the STS-3.

Based on these findings, in secondary analysis, Frantic Hopelessness minus Near Psychotic Somatization score was tested as a predictor of substantive current suicide attempt. Score on this measure ranged from −4 to 24 with mean(standard deviation) of 12.7(5.4). In binary logistic regression it remained a significant predictor, even after inclusion of gender and substance abuse, and BSI scores as above, with AOR 1.200 (p = 0.003). Receiver operator curve analysis showed fair differentiation between substantive suicide attempters and those who had ideation or very minor attempts only, with area under the curve of 0.724, p = 0.002. Optimal cut score of 13 coincided approximately with the mean, and yielded a sensitivity of 72.2% and a specificity of 60.5%. Score greater than or equal to 18, approximately one standard deviation above the mean, had 33.3% sensitivity and 80.9% specificity. See Figure 2.

### Predictive Validity

Of the eighteen subjects that consented to a follow-up interview 12 months after their initial ER interview and gave reliable information, two attempted suicide. Both had STS-3 total scores that increased from the initial interview. All but two subjects who did not attempt suicide had decreased STS-3 scores. Though these numbers are too small for reliable conclusions to be drawn, the difference between attempters and non-attempters in frequency of increased vs. decreased STS-3 scores was significant (Fisher’s exact test p = 0.03).

## Discussion

The present study aimed to validate a revised version of the STS, the STS-3, in an acute treatment setting – the psychiatric emergency room – which is often the point of first presentation of acute suicidality. Further, taking advantage of a larger sample size, and revision of the STS scale, we sought to refine our understanding of its internal structure.

We found that the scale had strong internal consistency, and factor analysis demonstrated three internally consistent, unidimensional, intercorrelated factors. These factors were

1. Frantic Hopelessness, comprising items describing entrapment, dread, and hopelessness, and correlating with BSI depression and anxiety subscales,
2. Ruminative Flooding, comprising items describing incessant and overwhelming rumination and a sense of one’s head bursting with uncontrollable thoughts, and correlating with BSI anxiety an paranoia subscales, and
3. Near Psychotic Somatization, comprising items describing strange somatic experiences and altered sensorium and correlating with BSI somatization and phobia subscales, and anti-correlating with the BSI depression subscale.

STS-3 total score, Frantic Hopelessness, and Ruminative Flooding scores correlated with severity of current suicide ideation, total STS-3 Frantic Hopelessness, and Ruminative Flooding subscale scores were higher among acutely suicidal subjects with history of suicide attempt than those without, and Frantic Hoplessness was a significant predictor of current substantive suicide attempts in both univariate and multivariate analyses, increasing odds of being an attempter by approximately 15–20%. Thus our study supports both the existence of an independent psychopathological, affective entity of the “suicide trigger state” and its relevance to acute suicidal risk.

Although two principal components were identified in the STS-2,three factors were found in the STS-3. Items for Factors 1 and 2, Frantic Hopelessness and Ruminative Flooding, were loaded analogously in both the STS-2 and the STS-3, however, the items for Near Psychotic Somatization, which loaded with ruminative flooding in STS-2, loaded as a separate third factor on the STS-3. Further, items describing a concretized experience of thought as creating head pressure, which had originally been interpreted as near psychotic somatization symptoms, loaded with ruminative flooding in the present analysis rather than with near psychotic somatization, while the somatic symptoms comprised by near psychotic somatization appear consistent with dissociative experiences. Thus replication of the current factor structure using confirmatory factor analysis is needed in further study of the scale.

The first STS-3 subscale, Frantic Hopelessness, was associated with depression and anxiety scores on the BSI. This is consistent with both the face-value relatedness of core content of the subscale items, and with multiple studies wherein depression, hopelessness, and anxiety were found to be the most common and strongest predictors of suicidal ideation, suicidal attempt, and suicidal intent [7], [22], [29]–[31]. Frantic Hopelessness, however, differs from simple anxiety and depression by its sense of urgency. In this, Frantic Hopelessness is similar to the Hendin’s concept of “suicide crisis” which is posited to precede suicide and is composed of factors including anxiety, desperation, hopelessness, abandonment, and loneliness [7]. One of the study participants likened the experience of frantic hopelessness to “being trapped inside an empty department store after hours and trying to get out, but all the doors are locked”. This explanation highlights the nature of Frantic Hopelessness as a heightened fear response to a perceived sense of entrapment and imminent doom.

The second component, Ruminative Flooding, was characterized by an uncontrollable onslaught of automatic and affectively-charged, negative thoughts associated with somatic symptoms in the head, such as headaches or head pressure. These findings are highly consistent with literature identifying robust associations between migraines, chronic daily headaches, and general headaches and increased risk of suicidal ideation, and current and past suicide attempts [32]–[38]. Even after controlling for risk factors including depression and other psychiatric disorders, Ilgen and colleagues [33] found strong associations between headaches and thoughts and suicidal behavior, underscoring our findings associating headaches with past SA.

In linear regression analyses, Ruminative Flooding most strongly correlated with BSI anxiety followed by BSI paranoia scales. This findingis consistent with current literature examining the relationship between cognitive distortion, maladaptive defenses, affective states and suicide [29], [35], [39]. Knock and Kazdin [29] demonstrated the presence of negative automatic thoughts to be significantly associated with suicidal ideation, suicidal intent, and the presence of a current suicide attempt. Even after controlling for depressed mood in the same study, negative automatic thinking remained strongly associated with a current suicide attempt. Further, Coleman [39] went on to suggest that the strong association of decreased maladaptive cognitions with decreased suicidal ideation supports further development of cognitive-behavioral interventions for suicidal patients.

The association of anxious symptomatology and somatoform experience of thoughts as creating head pressure with Ruminative Flooding is consistent with findings from Woolley’s study wherein anxiety acted as an important mediator in the association of headaches, with suicidal ideation and behavior. Woolley further noted that anxiety has been identified as a condition that may activate underlying suicidal propensities in depressed individuals [36]. Our findings are also consistent with Hendin’s reports of uncontrollable affects resulting in “fears of falling apart” which may be exacerbated under conditions of poor sense of self-continuity. [40], [41] Indeed, Hendin describes this as a “fragmentation” of the patient, finding it to be an experience strongly implicated in a suicide crisis or pre-suicidal state [7]. This fragmentation also closely resembles what Fawcett calls “psychic pain,” a state which involves agitation and anxiety and is also experienced immediately preceding a suicide attempt [42].

Near Psychotic Somatization, is characterized by the experience of somatic symptoms commonly associated with a panic-like dissociative state. Linear regressions revealed Near Psychotic Somatization to be robustly associated with the BSI somatization subscale. Unlike Ruminative Flooding’s head pressure, somatization in this component is indicative of somatic experiences characterized by unfamiliar bodily sensations felt all over the body, and especially involving the skin. However this factor appeared to be in fact somewhat protective against actual suicidal action in our sample. This is congruent with its negative correlation with BSI depression scores, but contrasts with much literature examining somatization [43]–[45], dissociation [46], and suicidality in at risk patient populations., It is consistent with the findings of others, however [47], [48], as well as findings for individual items on the STS-2 [8].

These inconsistencies may be a result of a global association of somatic and dissociative symptoms with greater severity of psychiatric pathology rather than association of suicidality with those symptom clusters, *per se*. Kemper and colleagues [49] found that patients with panic disorder or agoraphobia manifested more somatic concerns than patients with other anxiety disorders or patients with non-anxiety disorders. High rates of personality disorder have been found among patients with somatization disorder [50], and pathological dissociation was found to correlate with greater severity across multiple neuropsychological domains in borderline personality disorder [51].

Although limited by a small number of subjects reached for follow-up interview, our second hypothesis examining predictive validity of STS-3 for future SA was, albeit preliminary, confirmed. Our results indicate the likelihood of reattempting suicide is elevated for those sustaining higher STS-3 scores compared to those who exhibited a decrease in score at follow up. In other words, STS scores may predict whether or not an individual will re-attempt suicide within 12 months, suggesting that STS-3 or a similar scale could be used to monitor suicidal risk in the at risk patients. To our knowledge, these are the only results supporting the reliability and predictive validity of a measure designed to predict future suicide attempts in at risk individuals post-discharge.

The results of this study need to be considered in view of its limitations. Common to all studies employing self-report measures, it is possible that the data acquired from the subjects may be confounded by biased or inaccurate answers. Because the study was conducted in the emergency room of an urban community hospital serving a large homeless population, some of the participants’ answers may have been biased by hope of secondary gain from a hospital admission. Although the STS-3 does not directly ask about suicidal intent, it evaluates psychopathology in a way that is identifiable to a participant and thus is susceptible to over-reporting. Further, because of homelessness, a large number of participants could not be located for the follow up. Of those located, two provided conflicting information and had to be excluded. Thus, though the STS-3 appears to provide a measure of the syndrome’s severity during a three day period, the accuracy of this measure appears to be variable, and in particular to be at least in part dependent on subject’s interests in reporting their symptoms and/or trait disposition towards over or under reporting. Further, a number of potential participants admitted for serious suicide attempts did not consent to participation in the study, precluding data collection from those at highest suicide risk. Finally, the current report does not address the issue of whether the ‘Suicide Trigger State’ is a state condition or a trait susceptibility.

Finally, our finding of opposed effects of Frantic Hopelessness and Near Psychotic Somatization point to variability in the manifestation of this state and associated variability in its association with suicidal action. Thus, even if the measure can be taken at face value, given that the linkage between a suicide trigger state and actual attempt is probabilistic, further study is needed to determine the predictive value of identification and quantification of this state, and what additional predictive power, if any, it has in relation to other measures of suicidality. Moreover, as association does not imply causality, further research is required to elucidate the mechanism of linkage between a ‘trigger state’ and suicidal action. Such study could better inform therapeutic strategies for prevention.

Within its limitations, our study supports the existence of a distinct syndrome that may be experienced by individuals at risk for imminent suicide attempts. The syndrome of “suicide trigger state” combines frantic hopelessness, ruminative flooding, and near psychotic somatization, in a panic-like state, in agreement with literature supporting panic and anxiety as risk factors for suicide attempt [13], [15], [52]–[57] and is consistent with the existing literature on the pre-suicidal state of mind.
