# Correction: Emergency Room Validation of the Revised Suicide Trigger Scale (STS-3): A Measure of a Hypothesized Suicide Trigger State

**DOI:** 10.1371/annotation/dde14551-be58-4b7d-85e4-164563707b62

**Published:** 2012-10-01

**Authors:** Zimri S. Yaseen, Evan Gilmer, Janki Modi, Lisa J. Cohen, Igor I. Galynker

It has come to the author’s attention that, due to a data labeling error, “current substantive attempts” were in fact “current attempts by subjects with multiple (≥2) attempts”. Thus throughout the manuscript, “substantive attempts” should be read and understood as “repeat attempts.” This may suggest that elevated STS-3 scores relate to a characteristic state among persons with a trait tendency to take suicidal action.

In the study sample there were 10 (not 19) subjects who were current substantive attempters as defined in our methods. Analyses using substantive attempt as the dependent/outcome variable returned no significant results except for finding Near Psychotic Somatization subscale score approaching significance as a negative predictor of substantive SA (beta=-0.241, p=0.55) when regressed with scores on Frantic Hopelessness and Ruminative flooding subscales. When regressed without covariates beta was -0.186, p=0.093.

